# The performance of soluble CD163 as a non-invasive biomarker of liver damage in chronically HCV and HCV/HIV infected subjects

**DOI:** 10.1371/journal.pone.0270911

**Published:** 2022-07-07

**Authors:** Victoria Cairoli, Elena De Matteo, Paola Casciato, Beatriz Ameigeiras, María Victoria Preciado, Pamela Valva

**Affiliations:** 1 Laboratory of Molecular Biology, Pathology Division, Multidisciplinary Institute for Investigation in Pediatric Pathologies (IMIPP), CONICET-GCBA, Ricardo Gutiérrez Children’s Hospital, CABA, Buenos Aires, Argentina; 2 Liver Unit, Italian’s Hospital of Buenos Aires; CABA, Buenos Aires, Argentina; 3 Liver Unit, Ramos Mejía Hospital; CABA, Buenos Aires, Argentina; Ospedale San Raffaele, ITALY

## Abstract

Macrophage activation plays a key role in liver disease progression. Soluble CD163 (sCD163) is a specific macrophage activation biomarker useful for clinical estimating damage severity and predicting outcome in different liver conditions. sCD163 performance as a non-invasive marker of liver damage was evaluated in plasma samples at time of biopsy in 120 patients with different hepatic conditions (56 HCV, 20 HCV/HIV, 10 HBV and 34 MAFLD). sCD163 values were compared with those of healthy donors and analyzed related to histological damage. sCD163 together with other clinical parameters were used to create a logistical regression model to predict significant fibrosis. Only patients with viral hepatitis showed higher sCD163 values compared to the control group (HCV *p*<0.0001; HCV/HIV *p*<0.0001; HBV *p* = 0.0003), but no significant differences regarding fibrosis stages were observed. The proposed model predicts fibrosis severity using the logarithm sCD163 concentration, platelet count and age, it demonstrated to be a good marker for the HCV monoinfected group (AUROC 0.834) and an excellent one for the HCV/HIV co-infected group (AUROC 0.997). Moreover, the model displayed a diagnostic performance similar to FIB-4 in HCV cases and FIB-4 and APRI in HCV/HIV coinfected cases, and it even managed to correctly classify some cases that had been misclassified. The proposed model is able to determine, in a non-invasive way, the liver fibrosis stage of HCV and HCV/HIV patients, so after validation, it could be used in a complementary way in the clinical practice whenever APRI and FIB-4 failed to determine damage severity in HCV and HCV/HIV cases.

## Introduction

Currently, the increasing prevalence of chronic liver diseases is of great concern, given the high mortality and morbidity they present [1]. Sustained inflammation within the consequent fibrogenesis represents the basis of liver damage, hence the clinical manifestations of hepatitis vary from mild, self-limited inflammation to a severe form that may result in death [2].

Clinical evidence and animal models suggest that hepatic macrophages play an important role in several chronic liver diseases, since they are involved in liver homeostasis and development of liver inflammation and fibrosis [3]. Kupffer cells, the hepatic macrophages, represent up to 80–90% of the tissue macrophages [4], and are essential in the development and progression of liver diseases [5]. Macrophages develop flexible phenotypic responses to local environmental signals and produce a balanced array of cytokines with which they orchestrate the inflammatory response [6, 7]. Irrespective of the underlying pathology, during inflammation Kupffer cells produce cytotoxic substances such as reactive oxygen radicals causing liver necrosis, conversely, during fibrosis resolution the depletion of macrophages undermines matrix degradation and prevents recovery [8].

Macrophage-specific inflammatory markers are currently of great interest due to their role in inflammatory diseases [8], among them CD163 is considered as the hallmark of macrophages and monocytes [9]. CD163 acts as a surface hemoglobin-haptoglobin scavenger receptor which is constitutively shed from the cell surface into circulation as soluble CD163 (sCD163) during macrophages activation. In recent years sCD163, an easily accessible diagnostic and prognostic tool, seems to be of special value to monitor liver disease progression [9]. Remarkably high levels of sCD163, closely related to disease severity and outcome, are observed in acute liver failure, acute-on-chronic liver failure, and alcoholic hepatitis [8]. In the chronic HCV scenario sCD163 levels correlated with liver fibrosis, where cirrhosis displayed the highest values; besides a rapid decline in CD163 coincided with the decreasing in inflammation and fibrosis scores in response to direct acting antiviral (DAA), highlighting the role of macrophages [10–13]. On the other hand, lower sCD163 levels were found in Metabolic Associated Fatty Liver Disease (MAFLD), but this biomarker adds diagnostic information for the identification of patients with advanced disease [8]. Besides, sCD163 seems to be slightly elevated in obesity and it might be associated with obesity-related insulin resistance and the development of type-2 diabetes [8, 14–17]. In the HIV infection context, sCD163 may be a marker of macrophage-mediated disease progression since it is increased in these patients and then decreases after antiretroviral therapy, and correlates to viral load and CD4 T-cells [9, 18–22].

Concerning diagnosis and prognosis of most hepatopathies, liver biopsy currently remains as the gold standard method, but it is an invasive and risky procedure with still elevated costs that cannot be used as a tool to periodically monitor disease outcome [23]. Several noninvasive diagnostic strategies, namely serum markers and imaging methods, are now under study or being applied. Transient Elastography is widely accepted and used to measure liver stiffness; however, the equipment is not accessible and available at low income health centers. Consequently, there is still a need for new non-invasive and conceptually simple tests based on serological biomarkers that can be performed in any low-complexity laboratory to detect liver damage, monitor patients’ liver disease and/or develop preventive measures. In this context sCD163 seems to be a great candidate; nonetheless, its clinical routine use requires an international standardization, hence independent studies in different populations, pathologies and geographical regions are needed to validate its use.

In the present study, we evaluated sCD163 levels at a single time point in several well-characterized cohorts of subjects with: (i) chronic HCV (CHC), (ii) HCV/HIV, (iii) HBV infection (CHB) and (iv) MAFLD; and analyzed its diagnostic accuracy as a marker of liver damage that could be used in primary health care centers in developing countries. In turn, we analyzed sCD163 together with clinical and biochemical parameters to develop a new score for determining significant fibrosis.

## Material and methods

### Patients and samples

The study population involved a total of 120 subjects including 56 HCV monoinfected, 20 HCV/HIV coinfected, 10 HBV monoinfected, and 34 MAFLD; attending at the Hospital Italiano de Buenos Aires and Hospital JM. Ramos Mejía, Buenos Aires, Argentina. Formalin-fixed paraffin-embedded liver biopsies and concomitant plasma samples were obtained from each case.

In addition, plasma samples from 20 healthy adults with no clinical or biochemical parameters of liver disease or a known medical condition were also studied (median [range] 39.5 years [30–58]). All the individuals of the control group showed no serological markers compatible with hepatitis B virus, HCV or HIV infection.

CHC infection was defined by the presence of anti-HCV antibodies in serum and detectable HCV RNA in plasma samples in at least 2 separate occasions. HIV positive status was established by ELISA followed by a confirmatory Western blot assay. CHB was diagnosed by the presence of hepatitis B surface antigen (HBsAg) in serum samples for at least 6 months. Patients’ exclusion criteria were: other causes of liver disease, autoimmune or metabolic disorders, hepatocellular carcinoma (HCC). Related to treatment, only one HCV monoinfected and 5 HCV/HIV coinfected patients received direct-acting antivirals (DAA) therapy and the included samples were obtained at the end of treatment. HIV-infected patients were on stable ART for more than 1 year and had undetectable HIV viral load (< 20 copies/ml). CHB patients were naïve of treatment or free from it for at least 1 year.

Patients with MAFLD defined on the basis of hepatic steatosis established by liver echogenicity or with metabolic risk factors (i.e. obesity or metabolic syndrome) were enrolled. Those cases with other liver diseases such as autoimmune, genetic or endocrine diseases, HCC, HCV, HBV and/or HIV infection or alcohol ingestion greater than 30g/day for men and 20 g/day for women were excluded.

Serum alanine aminotransferase (ALT), aspartate aminotransferase (AST), platelet count, bodyweight, and height were obtained from clinical records. Aspartate aminotransferase-to-platelet ratio (APRI), aspartate aminotransferase-to-alanine aminotransferase ratio (AAR) and FIB-4 were calculated.

This study has the approval of the Institutional Review Board and the “Comité de Ética en Investigación del Hospital de Niños Ricardo Gutiérrez” in accordance with the Helsinki Declaration of 1975, as revised in 1983. A written informed consent was obtained from all the included adult patients after the nature of the procedure had been fully explained.

### Histological analysis

To minimize inter-observer errors, one pathologist reviewed all cases. Each biopsy from viral infected patients was categorized according to the modified Knodell scoring system (Histological Activity Index, HAI) [24], as minimal-mild (≤ 6) or moderate-severe hepatitis (> 6), and according to METAVIR [25] as significant fibrosis (≥2). Since METAVIR is more accurate to determine fibrosis stages, while modified Knodell classifies inflammatory activity and hepatitis with a deeper characterization of damage localization and severity, we selected each score to quantify the corresponding parameter. For MAFLD patients the histological diagnosis was performed using the NAFLD scoring system [26]. A 9-point scale (steatosis = 0–3; lobular inflammation = 0–3; ballooning = 0–2) was used to weigh activity grade; in this sense, a score ≥5 corresponds to “definitive NASH”, 3–4 to “borderline NASH”, and ≤2 to “not NASH or simple steatosis”. Meanwhile, the evaluation of fibrosis was based on a 6-point scale defined as 1a, b = mild (1a)/ moderate (1b) zone 3 perisinusoidal fibrosis; 1c = only portal fibrosis; 2 = zone 3 and portal/ periportal fibrosis, 3 = bridging fibrosis, 4 = cirrhosis. For all patient’s significant fibrosis was assumed when the fibrosis score was ≥ 2.

### Quantitative assessment of sCD163

Plasma samples were obtained from blood collected in EDTA tubes after centrifugation (3000 rpm, 10 minutes) and were stored at -80°C. Plasma sCD163 levels were determined by a commercial quantitative sandwich enzyme immunoassay technique (ELISA DuoSet Human CD163 R&D Systems (DY1607-05) + Ancillary Reagent Kit 2 (DY008), R&D Systems Inc, Minneapolis, USA) according to the manufacturer’s instructions. Patient’s samples were diluted 1/400 while control’s samples were diluted 1/200 in the Reagent Diluent buffer provided in the kit. All samples were assayed in duplicate. Plasma concentration for each marker was determined from standard curves. sCD163 concentration was expressed as mg/L.

### Statistical analysis

sCD163 was analyzed in relation to clinical and histological parameters of liver damage. Significant fibrosis was established by a newly developed score including sCD163 by means of a multiple ordered logistic regression analysis with METAVIR fibrosis score as the dependent variable and sCD163 as the explanatory one adjusted for age, gender, platelets, ALT, and AST (direct variables and continuous variables were logarithmically transformed). To identify the variables to be included in the new fibrosis severity score, a backward elimination was performed. The data set of individuals in each group according to pathology were randomly divided into a training set (75% of the cases) and a test set (25% of the cases) to develop and test the fibrosis severity new score.

GraphPad Prism version 5.01 (GraphPad Software, San Diego, CA, USA) was used. Outliers were examined by Grubb’s test. Normally distributed groups were analyzed with ANOVA or Student’s t-test and groups that didn’t meet this criterion were compared with Mann-Whitney U test or Kruskal–Wallis test, as appropriate. P values < 0.05 were considered significant. The receiver operating characteristic curves (AUROC) was applied to assess the diagnostic value, where an ideal test was defined by AUROC = 1 and a test of no diagnostic value by AUROC = 0.5. The cutoff value for the diagnosis was determined as the maximal value of the sum of the sensitivity and specificity.

sCD163 and the selected new score were compared to AAR, APRI and FIB-4.

## Results

### Studied population characteristics

Multiple independent cohorts were used for this study; therefore, groups were unmatched. Table 1 summarizes the demographic, clinical and histological features from the studied patients at the time of biopsy. For detailed information about each patient see S1 Table. In brief, median age for HCV, HCV/HIV and MAFLD groups were similar, but the HBV group median age was lower. The HCV/HIV and MAFLD groups showed male predominance. Most HCV (73.91%) and all of MAFLD cases presented elevated BMI (overweight or obesity). ALT at time of biopsy was elevated in the majority of cases (79% in HCV, 60% in HCV/HIV, 98% in MAFLD) except for HBV ones. HCV genotype 1 was predominant in both HCV mono- (58.93%) and HCV/HIV co-infected (65%) cohorts; HCV viral load were similar in both groups [HCV median (min.-max.) 1.09 10^6^ IU/ml (1.24 104–8.24 10^7^); HCV/HIV 3.20 10^6^ IU/ml (3.33 104–4.41 10^8^)] except for those HCV/HIV coinfected patients who received DAA therapy that displayed no detectable HCV viral load. Finally, HIV viral load was detectable in 3 cases and CD4% range was 10–63 (median 24%) [absolute value 424.3/ul (85.51–6732)].

**Table 1:** Clinical and histological patient features.

Factor	Patients			
	HCV	HCV/HIV	HBV	MAFLD
**Age (ys)** median (min.-max.)	52 (32–72)	49 (27–56)	36 (27–68)	49.5 (28–72)
**Gender** [male % (n/total)]	51.78 (29/56)	65.00 (13/20)	70.00 (7/10)	55.88 (19/34)
**Clinical and serological characteristics**				
**BMI**				
• Overweighed %(n/total)	63.04 (29/46)	30.77 (4/13)	NA	33.33 (11/33)
• Obese %(n/total)	10.87 (5/46)	15.38 (2/13)	NA	66.67 (22/33)
**Transaminases**				
**ALT (IU/l)** median (min.-max.)	76 (28–330)	55 (11–287)	38 (16–93)	78 (31–279)
• elevated %(n/total)	78.57 (44/56)	60.00 (12/20)	40.00 (4/10)	97.56 (33/34)
**AST (IU/l)** median (min.-max.)	61 (25–296)	50 (16–137)	26 (18–40)	17 (22–208)
• elevated %(n/total)	57.14 (32/56)	50.00 (10/20)	0.00 (0/10)	61.76 (21/34)
**Platelet (10** ^**9**^ **/L)**	179.5 (45.8–394)	198.5 (65–336)	227.5 (164–311)	216.50 (140–330)
**Histological characteristics**				
**Fibrosis**^1^%(n/total)				
• 0	3.85 (2/52)	-	40.00 (4/10)	67.65 (23/34)
• 1	32.69 (17/52)	63.16 (12/19)	30.00 (3/10)	17.65 (6/34)
• 2	28.85 (15/52)	5.26 (1/19)	30.00 (3/10)	11.76 (4/34)
• 3	28.85 (15/52)	21.05 (4/19)	-	2.94 (1/34)
• 4	5.76 (3/52)	10.53 (2/19)	-	-
**Hepatitis** ^2^ **% (n/total)**				
• Minimal	1.92 (1/52)	-	40.00 (4/10)	-
• Mild	17.31 (9/52)	31.58 (6/19)	40.00 (4/10)	-
• Moderate	63.46 (33/52)	47.37 (9/19)	10.00 (1/10)	-
• Severe	17.31 (9/52)	21.05 (4/19)	10.00 (1/10)	-
**Steatosis**%(n/total)				
• 0	51.920 (27/52)	84.21 (16/19)	50 (5/10)	-
• 1	19.23 (10/52)	15.79 (3/19)	20 (2/10)	14.71 (5/34)
• 2	25.00 (13/52)	-	20 (2/10)	26.47 (9/34)
• 3	3.85 (2/52)	-	10 (1/10)	58.82 (20/34)
**Lobular inflammation**%(n/total)				
• 0	-	-	-	20.59 (7/34)
• 1	-	-	-	58.82 (20/34)
• 2	-	-	-	20.59 (7/34)
• 3	-	-	-	-
**Ballooning** %(n/total)				
• 0	-	-	-	14.71 (5/34)
• 1	-	-	-	64.70 (22/34)
• 2	-	-	-	20.59 (7/34)
**NAFLD activity score** %(n/total)				
• ≤2	-	-	-	11.77 (4/34)
• 3–4	-	-	-	32.35 (11/34)
• ≥5	-	-	-	55.88 (19/34)
**n**	56	20	10	34

NA: not available. ALT: alanine aminotransferase. AST: aspartate aminotransferase. Normal ALT and AST levels were ≤40 and ≤42 IU/L, respectively when testing was done at 37°C. Normal Platelet range was 150–400 10^9^/L. BMI: Body Mass Index (kg/m^2^), normal weight (<25.0 Kg/m2), overweight (25.0–29.9 Kg/m2) and obesity (≥30 Kg/m2), in 10 HCV, 7 HCV/HIV, all HBV and 1 MAFLD patients BMI data were not available. ^1^Fibrosis according to METAVIR. 4 HCV and 1 HCV/HIV patients had a non-evaluable liver biopsy and therefore data about liver damage were not available. ^2^Hepatitis classification: minimal (HAI≤ 3), mild (HAI 4–6), moderate (HAI 7–12) and severe hepatitis (HAI>12). Steatosis Grade: score 0 (<5%cells), 1 (5–33%), 2 (33–66%) and 3 (>66%). Lobular inflammation: score 0 (0 foci), 1 (<2 foci), 2 (2–4 foci) and 3 (>4 foci). Ballooning grade: score 0 (none), 1 (few ballooning cells) and 2 (many cells/prominent cells); fibrosis stage: score 1 (a, b = mild (1a)/ moderate (1b) zone 3 perisinusoidal fibrosis; 1c = only portal fibrosis); 2 (zone 3 and portal/ periportal fibrosis), 3 (bridging fibrosis) and 4 (cirrhosis).

Regarding liver damage, HCV mono- and HCV/HIV coinfected patients presented more severe histological global findings. In particular, 80.77% of HCV biopsies revealed moderate/severe hepatitis and 63.46% significant fibrosis (34.61% advanced fibrosis including 3 cirrhotic cases); meanwhile 68.42% of HCV/HIV biopsies revealed moderate/severe hepatitis and 36.84% significant fibrosis (31.58% advanced fibrosis). On the other hand, HBV cases presented less severe signs of liver damage since 80% showed minimal/mild hepatitis and 70% showed no significant fibrosis, and, in fact, there were no advanced fibrosis cases. Finally, the histological review of MALFD cases depicted grade 3 steatosis, lobular inflammation grade 1 and ballooning grade 1 in most cases. Interestingly, fibrosis was absent in the majority of MAFLD cases (67.65%). In accordance with the report of the NASH Clinical Research Network classification, 55.88% of patients were classified as ‘definitive NASH’, 32.35% as ‘borderline NASH’, and 11.77% as ‘not NASH’.

### sCD163 plasma levels related to demographic and biochemical variables

**Fig 1:**
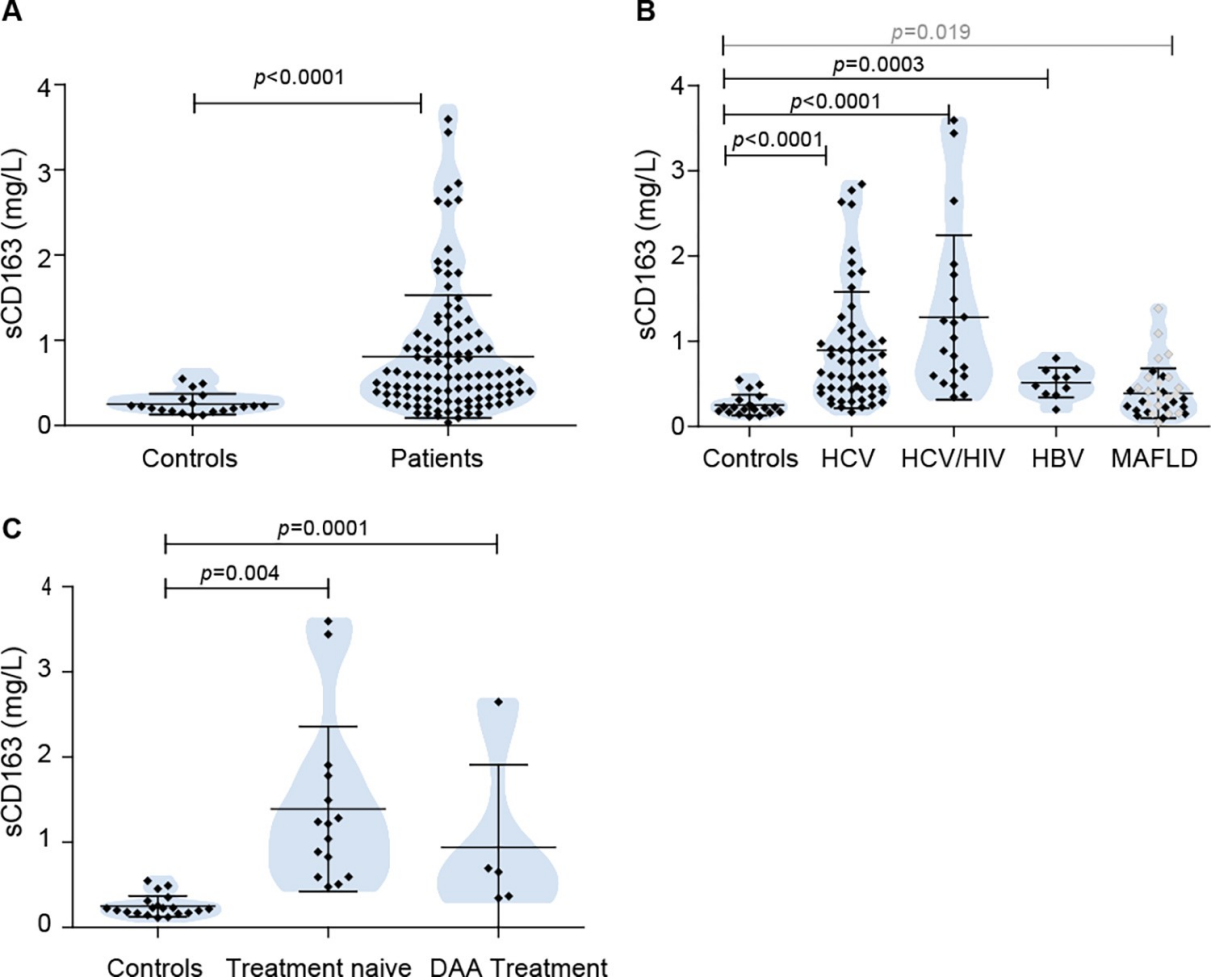
sCD163 in different conditions. A) Comparison of patients vs controls, B) detailed analysis according to disease etiology, and C) Comparison of treatment naïve vs. DAA treated HCV/HIV-coinfected patients. Grey dots represent NASH sCD163 values among MAFLD ones. Results are shown as median +/- SD.

sCD163 serum levels were elevated in patients when compared to controls [0.579 mg/L (0.034–3.596)] [0.221 mg/L (0.116–0.549)] (*p*<0.0001) (Fig 1A). However, the discrimination by etiologies showed significantly higher sCD163 levels only in the viral associated conditions [HCV 0.696 mg/L (0.168–2.884), *p*<0.0001; HCV/HIV 0.964 mg/L (0.345–3.596), *p*<0.0001; HBV+ 0.526 mg/L (0.199–0.802), *p* = 0.0003]. MAFLD patients displayed similar sCD163 levels to the control group [0.322 mg/L (0.034–1.374)], but, as expected, those patients classified as NASH displayed higher sCD163 values [0.403 mg/L (0.034–1.374), *p* = 0.019] (Fig 1B). HCV mono- and HCV/HIV-coinfected patients shared the highest sCD163 levels. Interestingly, in the group of HCV/HIV among DAA treated patients only one case presented a high sCD163 value (2.649 mg/L), the other ones depicted values lower than 0.700 mg/L although they did not reach uninfected control values (*p* = 0.004) [HCV/HIV treatment naïve 1.218 mg/L (0.478–3.596), HCV/HIV DAA treated 0.651 mg/L (0.345–2.649)] (Fig 1C).

sCD163 levels showed no age association in any group. When considering biochemical parameters, sCD163 levels negatively correlated with platelet counts in HCV cases (*r* = -0.469, *p* = 0.0003). While in HCV and HCV/HIV patients sCD163 levels positively correlated with ALT and AST (ALT-HCV *r* = 0.448, *p* = 0.0007; AST-HCV *r* = 0.589, *p* = 0.0001; ALT-HCV/HIV *r* = 0.541, *p* = 0.010; AST-HCV/HIV *r* = 0.587, *p* = 0.001), and sCD163 levels were found to be significantly higher in cases with elevated ALT (HCV *p* = 0.018, HCV/HIV *p* = 0.035) and AST (HCV *p* = 0.0001, HCV/HIV *p* = 0.027). Finally, sCD163 levels were similar for lean, overweight and obese patients in all the studied groups, therefore, BMI did not correlate with sCD163 levels in this series. In addition, sCD163 did not show correlation with CD4 count in HIV/HCV coinfected cases.

### sCD163 levels related to liver damage

**Fig 2:**
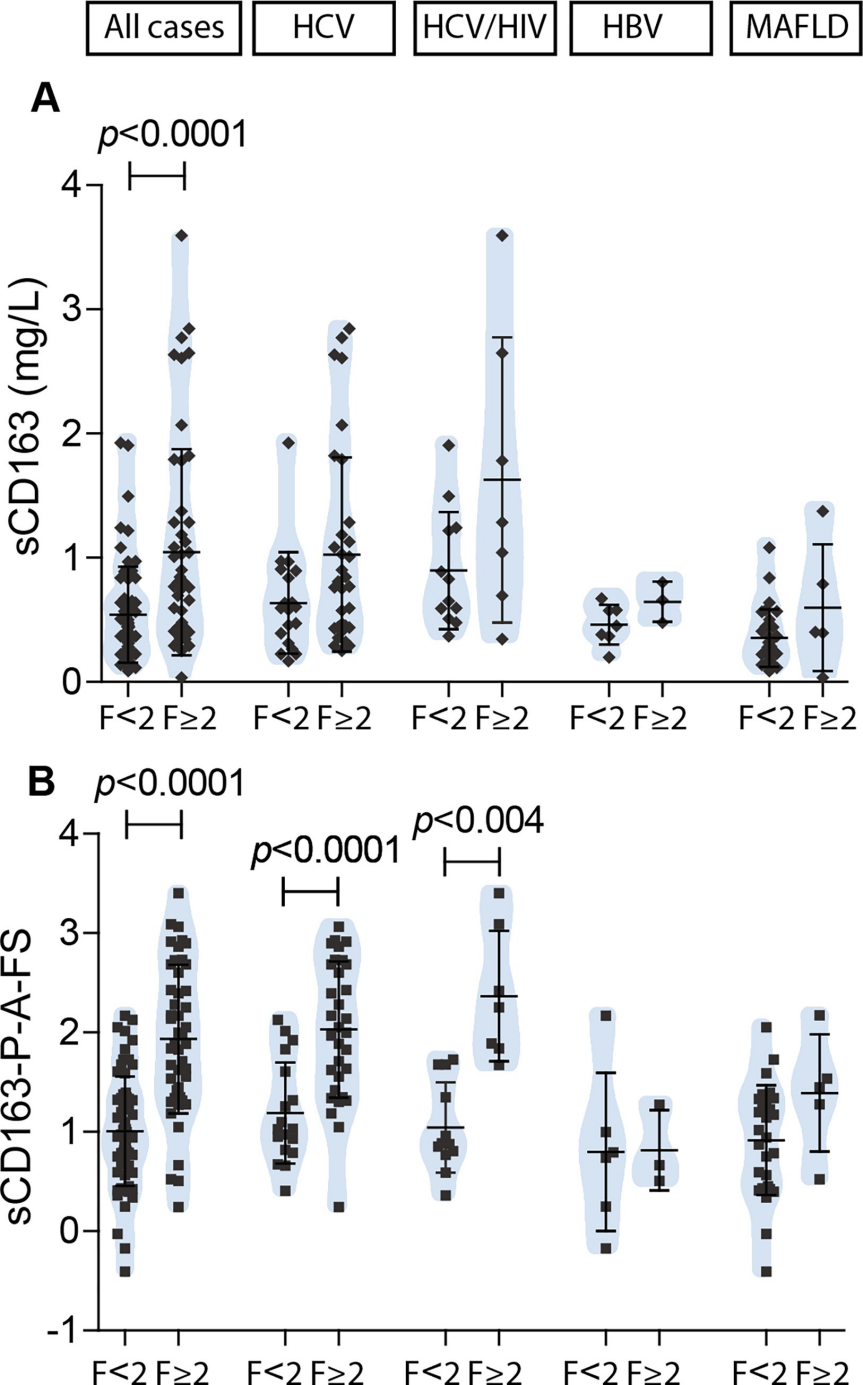
sCD163 comparative analysis according to fibrosis severity. A) sCD163 value, and B) proposed model. Results are shown as median +/- SD.

Those cases with significant fibrosis displayed higher sCD163 levels [0.778 mg/L (0.034–3.596)] than cases with a lower stage [0.459 mg/L (0.089–1.926)] (*p*<0.0001). Interestingly, in a detailed analysis according to disease etiology, no significant differences regarding fibrosis stage were observed in any of the studied groups; but patients with viral hepatitis (HCV, HCV/HIV and HBV) presented a profile with higher sCD163 values in those cases with significant fibrosis (Fig 2A).

**Fig 3:**
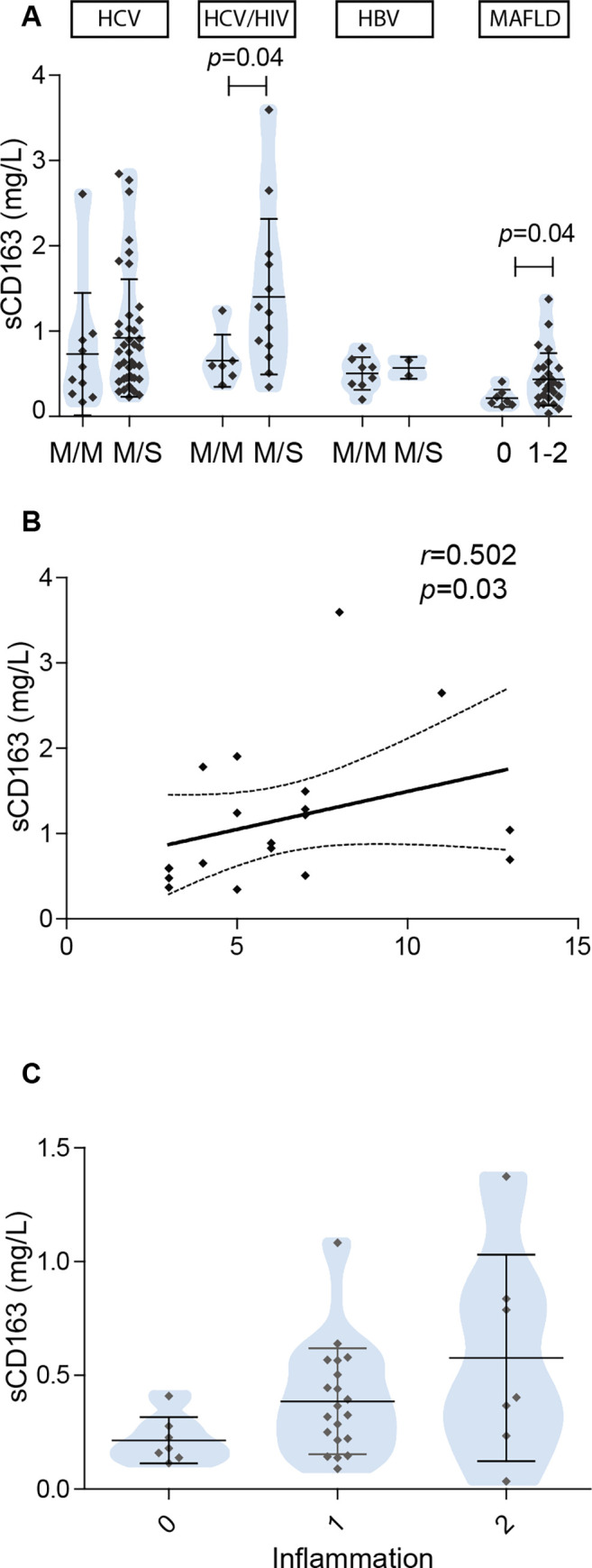
Comparative analysis related to inflammation. A) sCD163 related to hepatitis/inflammation severity. Viral infection cases: hepatitis severity was classified according to the Histological Activity Index, M/M: hepatitis minimal/mild (≤ 6), M/S: moderate/severe (> 6). MAFLD cases: inflammation was considered according to the NAFLD scoring system, B) correlation of sCD163 with inflammatory activity in HCV/HIV cases, and C) sCD163 vs inflammation in MAFLD cases. Results are shown as median +/- SD.

Regarding hepatitis severity, HCV mono- and HCV/HIV coinfected patients showed a profile of sCD163 with higher values in cases with moderate/severe hepatitis, but it turned out to be significant only in HCV/HIV patients (*p* = 0.04) (Fig 3A). In this latter group a correlation between sCD163 levels and the severity of inflammatory activity (*r* = 0.502, *p* = 0.03) was also observed (Fig 3B). Moreover, in MAFLD cases sCD163 levels were associated to inflammation since higher values were observed in those cases that presented inflammation (*p* = 0.04) (Fig 3A). Likewise, a profile with a progressive increase of sCD163 according to the severity of inflammation was observed in this group of patients (Fig 3C).

### sCD163 diagnostic performance

The diagnostic performance was only evaluated for those parameters that showed a significant association with histological injury. Table 2 shows the diagnostic accuracy of sCD163 sensitivity, specificity, and cut-off values.

**Table 2:** Diagnostic accuracy of sCD163 to determine histological staging.

	AUROC	95% CI	Cutoff	Sensitivity (%)	Specificity (%)
All cases- sCD163 for significant fibrosis	0.710	0.615–0.806	0.655	60.42	77.61
HCV/HIV- sCD163 for hepatitis severity	0.808	0.565–0.949	0.651	84.62	83.33
MAFLD- sCD163 for severe inflammation	0.751	0.574–0.883	0.227	66.67	85.71

AUROC, area under the receiver operating characteristic curve; CI, confidence interval.

When considering a biomarker as a less invasive test as good as a liver biopsy to evaluate liver damage, it should have an AUROC equal to or greater than 0.800. Under this assumption, sCD163 showed a good performance (AUROC: 0.808) related to hepatitis severity in HCV/HIV coinfected patients; however, despite the observed association between sCD163 and inflammation severity in MAFLD patients it did not display a good performance (Table 2).

### Development of a new score that includes sCD163 to predict significant fibrosis

As described above, with the aim of developing a new score including sCD163 as a predictor of significant fibrosis, a multiple ordered logistic regression analysis was performed. METAVIR fibrosis score was the dependent variable and sCD163, age, gender, platelet count, ALT, and AST (direct and logarithmically transformed variables) were the independent ones. The data set from all cases included in the training set were used to create the models. The coefficients (b) of the regression equations were used to calculate and examine all the possible predictive models. Three models that assume age, platelet count, and sCD163 as independent variables were obtained. Then their diagnostic values were tested and the following model was selected:

$$sCD163-P-A-FS=4.16+0.314\times {log}_{e}\left[sCD163(\frac{mg}{L})\right]-1.45\times {log}_{e}\left[platelet({\frac{10}{L}}^{9})\right]+1.31\times {log}_{e}[age(ys)]$$

Fig 2B shows the proposed model comparative analysis according to fibrosis severity and Table 3 summarizes the diagnostic accuracy of the proposed model to determine significant fibrosis. Briefly, the proposed model presented a good diagnostic value for discriminating significant fibrosis in the whole series which, in turn, proved to be good when applied and analyzed separately in the HCV monoinfected group and excellent in the HCV/HIV co-infected group. Interestingly, among HCV cases, 90% of the tested sample set was correctly categorized for significant fibrosis according to the cutoff value and only 10% was misclassified as false negatives (FN). Meanwhile, 100% of HCV/HIV cases of the tested sample set was correctly categorized. It should be noted that the performance of the model for discriminating significant fibrosis is better than sCD163 alone (AUROC 0.710 from Table 2 and 0.847 from Table 3).

**Table 3:** Diagnostic accuracy of the proposed model to determine significant fibrosis.

	AUROC	95% CI	Cutoff	Sensitivity (%)	Specificity (%)
All cases	0.847	0.758–0.936	1.265	88.57	72.73
HCV	0.834	0.706–0.961	1.279	91.30	64.71
HCV/HIV	0.997	0.885–1.048	1.785	83.33	100

AUROC, area under the receiver operating characteristic curve; CI, confidence interval.

### sCD163 performance compared to AAR, APRI and FIB-4

In order to compare the performance of sCD163 and the proposed model with other indexes widely used in clinical hepatology, AAR, APRI, and FIB-4 were calculated for each patient according to data availability.

Regarding fibrosis, AAR showed higher values in MAFLD cases (*p* = 0.03), with a good diagnostic performance. APRI showed higher values in HCV mono- (*p* = 0.007) and HCV/HIV coinfected cases (*p* = 0.02), but with good diagnostic accuracy only in HCV/HIV coinfected patients. Finally, FIB-4 showed significantly increased values in HCV mono- and HCV/HIV coinfected patients with significant fibrosis (HCV *p* = 0.0001, HCV/HIV *p* = 0.0005) as well as in MAFLD patients both when considering them as a whole group and as a NASH subgroup (MAFLD *p* = 0.0084, NASH *p* = 0.008). FIB-4 showed a good diagnostic value to predict significant fibrosis in all groups (AUROC ≥ 0.800). Results are summarized in Table 4.

**Table 4:** AAR, APRI and FIB-4 AUROC to determine significant fibrosis.

	AUROC	95% CI
**HCV**		
APRI	0.732	0.589–0.874
FIB-4	0.866	0.759–0.974
**HCV/HIV**		
APRI	0.867	0.690–1.043
FIB-4	0.950	0.843–1.050
**MAFLD**		
AAR	0.807	0.665–0.948
FIB-4	0.877	0.729–1.027
**NASH**		
FIB-4	0.910	0.738–1.084

AUROC, area under the receiver operating characteristic curve; CI, confidence interval.

**Fig 4:**
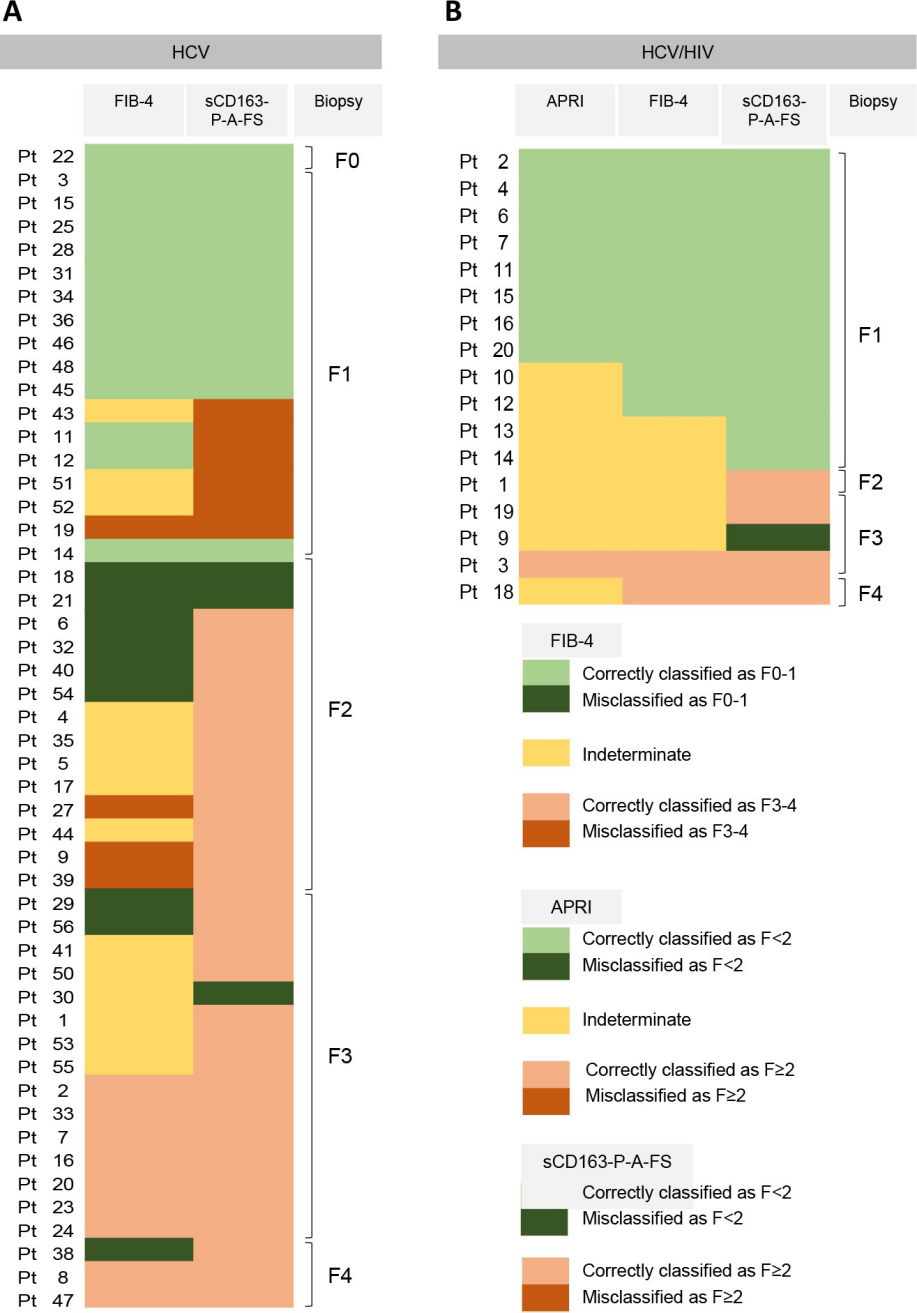
Evaluation of the concordance/discordance of the results obtained by APRI, FIB-4 and the proposed model. A) FIB-4 and the proposed model in HCV cases, B) APRI, FIB-4 and the proposed model in HCV/HIV cases. Low fibrosis stage: light green indicates cases correctly classified; dark green indicates cases misclassified. Yellow indicates indeterminate. High fibrosis stage: light red indicates cases correctly classified, dark red indicates cases misclassified.

It is important to highlight that in HCV patients the performance of FIB-4 was similar to the proposed model (Tables 3 and 4). Considering the reported FIB-4 cut-off values for individuals with HCV (<1.45 no significant fibrosis, 1.45–3.25 indeterminate, >3.25 for F3-4), 23/50 cases would have been diagnosed with F0-1, and 13/50 would have been diagnosed with F3-4. The proposed model results were concordant with those of FIB-4 in 24/36 cases, but interestingly 3/24 cases were misclassified by both methods. From those 12/36 cases with discordant results, 10/36 cases were only correctly classified by the proposed model. Finally, according to the FIB-4 cut-off values 14/50 cases would have been indeterminate, but the proposed model correctly classified all F2 and 5/6 F3 (Fig 4).

On the other hand, in the HCV/HIV coinfected subset the proposed model had higher AUROC than APRI and FIB-4 (Tables 3 and 4). Considering the APRI cutoff (<0.5 no significant fibrosis, 0.5–1.5 indeterminate, >1.5 for F≥2) 8/17 would have correctly been diagnosed with F0-1 and 1/17 with F≥2 but 8/17 would have been indeterminate. The proposed model was able to solve 7/8 indeterminate cases. In a similar way, 5/17 cases were classified as indeterminate with FIB-4, but 4/5 were correctly classified with the proposed model (Fig 4).

### Diagnostic performance of the studied scores to predict hepatitis severity

Since AAR, APRI, and FIB-4 include transaminase values that, at some point, could be considered a reflection of hepatic inflammation, an unconventional analysis was performed in this series to evaluate whether these indexes were associated with the severity of hepatitis. In this sense, in HCV/HIV coinfected patients elevated APRI levels were associated with moderate/severe hepatitis (*p* = 0.01) with a good diagnostic value (AUROC 0.894, Se 100%, Sp 66.67%). On the other hand, as mentioned above, sCD163 also showed good performances for hepatitis severity prediction in HCV/HIV coinfected patients (Table 2). Finally, in this group of coinfected patients, the diagnostic effectiveness of sCD163 was evaluated when each parameter was applied consecutively or simultaneously with APRI. However, in neither case this strategy improved the performance of APRI as a single marker.

## Discussion

Circulating macrophage-derived biomarkers from biological fluids that reflect their activation in tissues are highly sought after. In recent years, the diagnostic and prognostic value of sCD163 has been evaluated in various conditions and has proven to be especially valuable in liver diseases [9, 27].

sCD163 is currently determined either with in-house or commercial ELISAs approved for research use only. Besides, sCD163 is thoroughly applied in clinical samples in several reports. So there is still a remaining challenge of standardization of these assays since its lack leads to variable sCD163 levels reported across different studies, an issue that must be resolved before the biomarker could be applied in the routine clinic [9, 28]. In this sense our cohort, although it is small, contributed to the enlargement of the studied series particularly including cases from Latin America which are underrepresented.

In the studied cohort, sCD163 values were higher in samples from individuals with viral liver diseases compared to controls, even in the HBV group, which was represented by an extremely low number of cases. In the whole cohort, we observed that sCD163 values were independent from age, although it was one of the parameters selected by our model to assess the severity of fibrosis. BMI did not condition sCD163 levels since similar values were observed for lean, overweight, and obese patients. When analyzing sCD163 in the context of other biochemical markers, sCD163 levels negatively correlated with platelet count in HCV cases while sCD163 levels positively correlated with transaminase both in HCV and HCV/HIV patients. However, the association between sCD163 with clinical parameters is contradictory and it seems to depend on the studied group. Moller HJ et al. observed that sCD163 increases significantly with age, but Fjeldborg K et al. observed that sCD163 values were independent from age and gender [17]. For its part, NOBIDA biobase described a significant correlation of sCD163 with the BMI, lipid metabolism biochemical parameters, ALT and GGT. Other studies showed that sCD163 was elevated in obese subjects and that sCD163 was associated with insulin resistance [9, 17, 27].

Many authors describe that in chronic viral hepatitis sCD163 reflects the innate immune activation, which is the pathogenic basis of disease progression and, in turn, that in those cases with similar stages of fibrosis and inflammation,higher sCD163 levels could reflect a more aggressive inflammatory and fibrogenic environment [13, 27, 29, 30]. HCV chronic infection is associated with metabolic disorders in which monocyte activation and inflammation can play a major role; besides it is well known that coinfection with HIV accelerates HCV related liver fibrosis [31]. In our study, sCD163 plasma levels were increased in HCV and HCV/HIV coinfected patients according to the results of Mascia C et al. [31]. These two groups presented the highest sCD163 values of the entire cohort and they displayed a profile tending to higher sCD163 levels in cases with more severe inflammation and fibrosis as previously shown by other authors [27, 32].

It has been stated that sCD163 levels mirror the successful viral therapy displaying a significant decrease after treatment; however, sCD163 values do not fully normalize post-ART or post–HCV DAA therapy which may reflect the cooperative force of HIV and HCV to elevate sCD163, as a consequence of the interactive and additive effects in HIV/HCV coinfection [22, 32–36]. Despite the low number of treated cases, this behavior was observed in our cohort since DAA treated HCV/HIV patients showed low sCD163 levels, although they did not reach those values observed in uninfected controls.

The pathogenesis of MAFLD is considered as multifactorial and its most severe manifestation, NASH, presents severe alterations in the innate immunity, specifically in macrophages. Different studies in morbidly obese NASH adults set for bariatric surgery, showed that sCD163 increases according to the progression of the disease from simple steatosis to NASH and also according to fibrosis progression. Following surgery, sCD163 levels decrease 30–40% in association with liver enzymes and with the improvement in insulin sensitivity [37, 38]. Remarkable, Ragab H et al. described that serum sCD163 was augmented in Non-Alcoholic Fatty Liver Disease (NAFLD) cases compared to healthy controls, but it actually showed significantly higher levels in the obese- and non-obese diabetic patients as compared with lean healthy subjects. Hence, the authors proposed sCD163 as a diagnostic marker for NAFLD; however, it did not correlate with the NAFLD fibrosis score which prevented it to predict fibrosis severity. Our results partially reflected these observations, since MAFLD cases displayed a profile with higher sCD163 levels compared to controls, but these differences were statistically significant only in the NASH subgroup. sCD163 levels showed no correlation with lean, overweight and obese conditions or with fibrosis severity. Nevertheless, sCD163 levels seem to reflect the role of macrophages in MAFLD pathogenesis, as they were associated with inflammation and showed a progressive increase according to inflammation severity. While this might suggest its possible usefulness as an inflammatory severity follow-up marker, sCD163 did not demonstrate to be good enough (AUROC: 0.751). Perhaps the study of a larger cohort could allow us to confirm other authors’ suggestion about its capability for assessing damage progression.

An interesting point to consider is that, although the integrated analysis of the whole series showed significantly increased sCD163 values in relation to fibrosis severity, this fact did not hold true when they were analyzed individually in each pathology. Kazankov K et al. described that the association between sCD163 and fibrosis is better when it is adjusted for multiple biochemical and clinical parameters [13]. Thus, they developed two noninvasive models, each one for predicting fibrosis in HCV or HBV infected patients. The Kazankov K et al. [13] article inspired us to develop our own algorithm to analyze our cohort since, unfortunately, we do not have the HOMA-IR to be able to apply the model developed by Kazankov K et al. to patients with HCV in our series, and when applying the model developed for HBV to our cases, it showed no association with the severity of fibrosis. Our proposed model demonstrated a good diagnostic value for discriminating significant fibrosis in the HCV monoinfected group, and it had an excellent performance in the HCV/HIV coinfected group. Moreover, our model displayed a diagnostic performance similar to FIB-4 in HCV cases in addition to FIB-4 and APRI in HCV/HIV coinfected cases, and it even managed to correctly classify some cases that had been classified as indeterminate by these widely clinically used indexes. Therefore, this proposed model, after proper validation, could be used in a complementary way in the clinical practice whenever APRI and FIB-4 could not determine damage severity in patients with HCV and HCV/HIV. Interestingly, when we applied the proposed model to predict fibrosis in MAFLD patients, its performance did improve neither APRI nor FIB-4 fibrosis staging power, so in our series the later are still the algorithms of choice for these patients.

When considering hepatitis, sCD163 showed a good performance for determining severity in HCV/HIV coinfected patients; however, they did not improve the APRI performance. It would be interesting to include an inflammation marker such as plasmatic IL-6 in the new developed model to strengthen its power for hepatitis severity staging a parameter of no less importance.

Ultimately, it should be born in mind that the present study has certain limitations. First, the number of enrolled cases is quite limited, so in order to address this pitfall we tried to validate our proposed model in an independent dataset. We tested the proposed model in raw data from 47 HCV and 90 HCV/HIV patients from the Liver Disease Tissue Repository (LDTR) and the AIDS Clinical Trials Groups (ACTG-A5071), respectively, kindly shared by Dr. Alatrakchi [32]. Interestingly, in this data set the model also displayed the association with fibrosis severity previously observed in our cohort. Second, the proportion of cases included in each stage of damage is not completely equivalent in our studied cohort. Unfortunately, the addition of the dataset from Dr. Alatrakchi could not balance the fibrosis stage distribution within the cohort. So a follow-up prospective study would be important to validate our findings. In any case, sCD163 proved to be an easily measurable marker that could be evaluated in low complexity laboratories. On the other hand, the proposed model can be easily calculated from routinely determined clinical and biochemical variables plus sCD163 concentration, an easily measurable parameter that does not require much technical complexity.

In conclusion, macrophages are particularly activated in chronic viral hepatitis, and their activation would be quantifiable by measuring sCD163 levels. Our proposed model is simple and includes on the one hand readily available parameters like age and platelets and, on the other hand, an indicator of macrophage activation, a marker of the pathophysiology of the disease. This research adds valuable results to previous studies that propose the use of sCD163 as a marker of liver damage and; moreover, suggests a new model that could be easily applied to elucidate those indeterminate cases. This analysis should be considered as a pilot study that contributes to the still pending validation by testing samples of an underrepresented geographic region. This study provides the opportunity for future discussion in the scientific community about the incorporation to the clinical practice of this proposed model including sCD163 as a marker of significant fibrosis in HCV monoinfected and HCV/HIV co-infected patients.

## Supporting information

S1 Table Clinical, biochemical and histological patient features. (XLSX)
